# A Case for Help

**Published:** 1888-11-17

**Authors:** A. E. Young

**Affiliations:** Ironmongers' Almshouses


### A CASE FOR HELP.

I have to thank you for so kindly publishing my letter *re* Charles Bridgett. I have already the good news to tell you that I received £3 last night from Lady Rothschild, which is at once most liberal and most kind of her. Success now seems very nearly achieved in this little undertaking, and I believe the poor patient will be much cheered in consequence.

A. E. Young.

Ironmongers' Almshouses, November 11th, 1888.

